# PDC-Net: parallel dilated convolutional network with channel attention mechanism for pituitary adenoma segmentation

**DOI:** 10.3389/fphys.2023.1259877

**Published:** 2023-08-30

**Authors:** Qile Zhang, Jianzhen Cheng, Chun Zhou, Xiaoliang Jiang, Yuanxiang Zhang, Jiantao Zeng, Li Liu

**Affiliations:** ^1^ Department of Rehabilitation, The Quzhou Affiliated Hospital of Wenzhou Medical University, Quzhou People’s Hospital, Quzhou, China; ^2^ Department of Rehabilitation, Quzhou Third Hospital, Quzhou, China; ^3^ College of Mechanical Engineering, Quzhou University, Quzhou, China; ^4^ Department of Thyroid and Breast Surgery, Kecheng District People’s Hospital, Quzhou, China

**Keywords:** pituitary adenoma, image segmentation, U-Net, parallel dilated convolutional, residual connections

## Abstract

Accurate segmentation of the medical image is the basis and premise of intelligent diagnosis and treatment, which has a wide range of clinical application value. However, the robustness and effectiveness of medical image segmentation algorithms remains a challenging subject due to the unbalanced categories, blurred boundaries, highly variable anatomical structures and lack of training samples. For this reason, we present a parallel dilated convolutional network (PDC-Net) to address the pituitary adenoma segmentation in magnetic resonance imaging images. Firstly, the standard convolution block in U-Net is replaced by a basic convolution operation and a parallel dilated convolutional module (PDCM), to extract the multi-level feature information of different dilations. Furthermore, the channel attention mechanism (CAM) is integrated to enhance the ability of the network to distinguish between lesions and non-lesions in pituitary adenoma. Then, we introduce residual connections at each layer of the encoder-decoder, which can solve the problem of gradient disappearance and network performance degradation caused by network deepening. Finally, we employ the dice loss to deal with the class imbalance problem in samples. By testing on the self-established patient dataset from Quzhou People’s Hospital, the experiment achieves 90.92% of Sensitivity, 99.68% of Specificity, 88.45% of Dice value and 79.43% of Intersection over Union (IoU).

## 1 Introduction

As an important medical imaging technology, magnetic resonance imaging (MRI) has been widely used in the examination of pituitary adenoma because it can display the anatomical information of soft tissues (Gavirni et al., 2023). At present, the analysis and processing of medical images mainly rely on the visual discrimination of brain MR Images by clinicians. This process is not only inefficient and time-consuming, but also has significant subjective limitations. In addition, it may not be replicable. With the development of computer science and artificial intelligence, the introduction of multidisciplinary medical imaging technology can design accurate treatment plans and give more reliable factors to the diagnosis. Therefore, the research on the segmentation method of pituitary adenoma MR image has important theoretical significance and practical application value for the development of modern medical informatization and intelligent computing of medical image.

Over the past few decades, various medical image segmentation algorithm have been presented, which can be broadly grouped into thresholding (Jain and Singh, 2022; Rawas and El-Zaart, 2022), watershed (Mohanapriya and Kalaavathi, 2019; Sadegh et al., 2022), clustering (Xu et al., 2022; Zhou et al., 2022), conditional random field (Sun et al., 2020; Li et al., 2022), dictionary learning (Yang Y Y et al., 2020; Tang et al., 2021), graph cut (Gamechi et al., 2021; Zhu et al., 2021), region growing (Rundo et al., 2016; Biratu et al., 2021), active contour (Dake et al., 2019; Shahvaran et al., 2021), quantum-inspired computing (Sergioli et al., 2021; Amin et al., 2022), computational intelligence (Vijay et al., 2016; Zhang et al., 2022). These traditional methods rely on developers to design algorithms for specific applications. They are highly interpretable, require few hardware devices, and do not require extensive data annotation. However, some algorithms or models need complex parameter tuning during in the process of implementation, so their generalization ability and robustness will be affected for specific segmentation problems.

Currently, with the development of computer hardware, deep learning methods have brought tremendous changes to the field of medical image segmentation, especially the convolutional neural networks (CNNs) framework. Among these new CNNs architectures, the most famous is U-Net proposed by Ronneberger et al. (2015). The main innovation lies in the rational design of the upper and lower sampling layer and the jump connection, so that the spatial information lost in the contraction path and the spatial information lost in the expansion path can be fused to produce a higher resolution local output. Since then, many scholars have integrated many theories into the U-Net framework according to the application requirements, so that U-Net system has been greatly expanded. For example, Lu et al. (2021) proposed a WBC-Net that automatically screens for white blood cells from smear images. Firstly, the residual network is added to deepen the network structure and enhance the ability of feature extraction. Then, WBC-Net introduces a mixed jump path, which can better fuse the features of different levels in the encoding structure and decoding structure, and thus improve the segmentation accuracy. According to the structural characteristics of vestibular, Zhang et al. (2021) constructed a deep learning network architecture based on supervision. Based on an encoder-decoder network, the model fuses the characteristic information of different receiving domains and attention mechanisms, which greatly improves the accuracy of network segmentation. AboElenein et al. (2022) proposed IRDNU-Net for the automatic segmentation of brain tumours in MRI images. The network used convolution cores of different sizes in the encoding path and decoding path, then reduced the dimension of the channel through 1 × 1 convolution to reduce the computational complexity, and finally carried out superposition and output to the next layer of the network. Due to the different sizes of the convolution kernel, the network can effectively extract the image features of different regions. Zhang et al. (2022) used DenseNet blocks to replace the convolution blocks of the original U-Net for the segmentation of skin lesions. This operation makes the current layer not only related to the output of the previous layer but also dependent on all previous layers, significantly promoting the propagation of the gradient.

Although the above deep learning algorithms have achieved good results in medical data, there is still a big gap between it and clinical application. This primarily has two reasons. First, the generalization ability of most deep neural network models is limited, and their performance in the real data of hospitals will be greatly reduced, mainly due to the differences of individual patients, the diversity of data types and the difficulty in the characterization of diseases. Second, in the current network architecture, the lack of capturing multi-scale context information leads to the deficiency of feature learning ability.

Inspired by the above thoughts, we propose a new U-Net architecture for pituitary adenoma segmentation. Based on the classical encoder-decoder framework, our network contains three core strategies: parallel dilated convolutional module, channel attention mechanism and residual connections. The combination of these strategies provides good segmentation ability for pituitary adenoma, and the Dice value, Intersection over Union and F1-score reach 88.34%, 79.25%, and 91.52%, respectively. The contributions of this study can be summarized as follows:

(1) A parallel dilated convolutional neural network based on U-Net architecture is built for pituitary adenoma segmentation.
(2) Replacing the traditional standard convolution block with PDCM, to extract more abundant multi-level feature information. Furthermore, we integrate the channel attention mechanism into PDCM to further strengthen the capability of the network.
(3) The introduction of residual connections at each layer can eliminate gradient disappearance and improve segmentation precision by constructing deeper networks.

## 2 Materials and methods

### 2.1 Overview of the network

**FIGURE 1:**
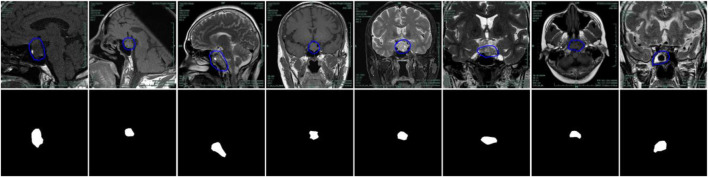
Examples of problems in segmenting pituitary adenoma from MR images. The first row: original images. The second row: the gold standard sketched by experts.

**FIGURE 2:**
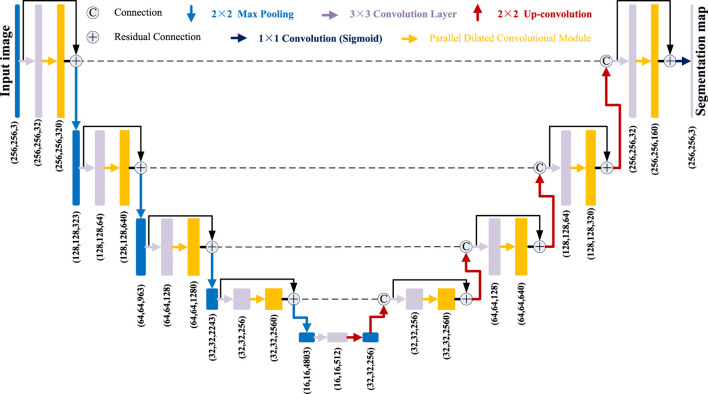
Network architecture of our PDC-Net.

As shown in Figure 1, the pituitary adenoma lesions that need to be segmented take up a small part of the entire image, far less than the background area. The dataset consists of 38 brain MRI scans of patients. There are 1,200 cases in the training set, 400 cases in the validation set, and 400 cases in the test set. The annotation work was manually outlined by three medical experts, and then segmented and annotated by five computer annotators using Lableme. After the dataset was generated, it was finally reviewed by three medical experts to obtain the final dataset (Pusparani et al., 2023). Another problem is the shape is irregular and the contrast with its surrounding tissue is not obvious. For the above difficulties, the architecture of PDC-Net is derived from the U-Net network, as shown in Figure 2. Our PDC-Net consists of two parts: an encoder module and a decoder module. The encoder branch consists of five layers, each of which contains a basic convolution operation and a PDCM, connected by residuals. After that, a 2 × 2 max-pooling operation is used for downsampling. In each downsampling process, the image size will be reduced to half of the original size, and the number of feature channels will be doubled. Accordingly, in the decoder branch, an equal amount of upsampling processing is performed to obtain an output image with the same size as the input image. During the upsampling process, the image size is doubled using deconvolution operations and connected with the features of the same resolution from the encoder path, followed by a convolution layer and a PDCM layer. In this network structure, the application of a large number of batch normalization layers (Jha et al., 2021) and ReLU activation function (Bai et al., 2021) can not only accelerate the training speed, but also improve the network generalization performance and stability. In addition, the DropBlock strategy is introduced in the convolutional layer to prevent network over-fitting. To generate the final segmentation result, the 1 × 1 convolution and sigmoid functions are used at the last layer to get the desired categories. Detailed introduction can be found in the following subsections.

### 2.2 Parallel dilated convolutional module

In the traditional convolutional neural network, continuous convolution and pooling operations will lead to a decrease in image resolution and the loss of spatial structure information, which has a great impact on the task of medical image segmentation, and directly lead to the blurred boundary and pixel classification errors after segmentation. With the appearance of atrous spatial pyramid pooling (ASSP) structure (Chen et al., 2018b), the above problems can be solved well, and many optimized models are proposed continuously (Ni et al., 2021; Xie et al., 2021; Lan et al., 2022; Liang et al., 2022). By connecting convolution layers with different expansion rates in parallel, ASSP can alleviate the problem of spatial information loss caused by downsampling, and enlarge the receptive field without increasing the amount of computation, to obtain more regional information. However, these methods ignore the boundary information when fusing multi-scale features, which is not effective for small objects. Therefore, a parallel dilated convolutional module integrated with the channel attention mechanism is proposed.

**FIGURE 3:**
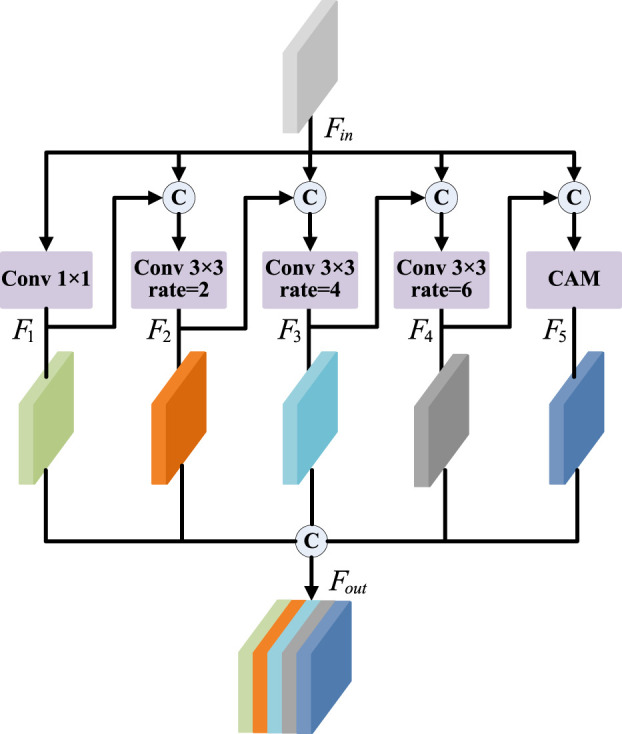
Structure of PDCM.

**FIGURE 4:**
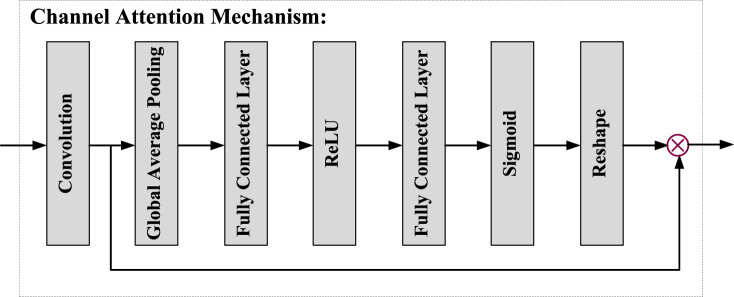
Structure of CAM.

As shown in Figure 3, the PDCM consists of five parallel channels: four convolution layers (with different dilated rates) and a channel attention mechanism. Firstly, the first branch is a 1 × 1 convolution, which aims to maintain the original receptive field while reducing the number of channels in the feature map. The second to fourth branches use convolution of different dilated rates to obtain different receptive fields, to fuse the feature information of different scales. However, due to the very small proportion of pituitary tumour region in the whole MR image, too large dilated rate will cause discontinuity of the receptive field, so we set the dilated rate from {6,12,18} to {2,4,6}. The fifth branch is the channel attention mechanism, as shown in Figure 4. CAM module obtains the weight information on the channel by pooling, channel convolution, and sigmoid of the input feature, so it can not only retain the underlying features of the target boundary information, but also obtain more context information. In addition, we do not fuse five different scales of information simply. We incorporate the feature output from the previous branch into the input feature of the next branch, and then perform the scale transformation. For example, assuming that $F_{in}$ is the input feature, and its output is $F_{1}$ after the first branch. Then, we will take ( $F_{1}+F_{in}$ ) as the input of the second branch, followed by a convolution operation with a dilated rate of 2. Similarly, the inputs of the third to fifth branches are ( $F_{2}+F_{in}$ ), ( $F_{3}+F_{in}$ ) and ( $F_{4}+F_{in}$ ), respectively. Finally, the output feature $F_{out}$ of PDCM is ( $F_{1}+F_{2}+F_{3}+F_{4}+F_{5}$ ). By using this connection option, the network can get a feature map with additional dimensions and scale context information, which will help it classify border pixels more accurately.

### 2.3 Residual connections

**FIGURE 5:**
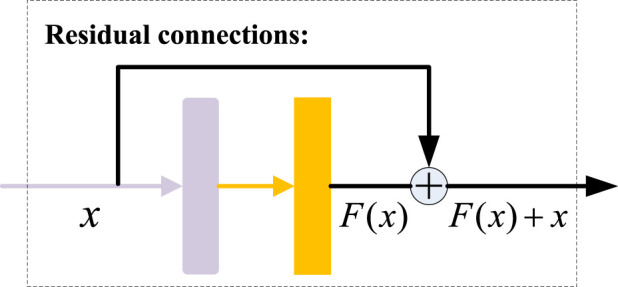
Residual connections.

The experimental results show that the performance of a deep network does not increase consistently with the increase in the number of layers. When there are too many layers in the network, the phenomenon of gradient disappearing and dispersion may occur. Therefore, to improve the stability of the network in the deep learning training process and alleviate the problem of gradient disappearance, the residual connections are introduced into our model. As shown in Figure 5, we add a shortcut branch outside the main network, and then connect the main branch input x with the main branch output $F\left(x\right)$ . After adding the residual connections, we could make full use of each feature graph before and after convolution, which solved the degradation problem and greatly improved the learning ability of the network.

### 2.4 Loss function

Due to the small data set, different lesion sizes, uneven foreground and background, it is easy to produce class imbalance problem. Dice Loss function (Arora et al., 2021; Liu et al., 2021; Phan et al., 2021) can judge from a global perspective and reduce the influence of the foreground image, which is adopted to train the network in this paper, and its calculation formula is defined as follows:

$$L_{Dice}=1-\frac{2\sum_{i=1}^{N}y_{i}{\hat{y}}_{i}}{\sum_{i=1}^{N}y_{i}^{2}+\sum_{i=1}^{N}{\hat{y}}_{i}^{2}}$$ (1)

where $L_{Dice}$ is the Dice loss, N denotes the number of pixels, ${\hat{y}}_{i}$ represents the prediction probability that the ith pixel belongs to the lesion, $y_{i}$ is the actual label of the ith pixel.

## 3 Results and discussion

All models were based on NVIDIA Quadro RTX 6000 GPU memory of 24 GB, CUDA 11.0.2, cuDNN v8.0.4, and TensorFlow 2.4.0. These models were trained with the batch size set to 16 and the epochs set to 300. In addition, the Adam optimizer (Ji and Wu, 2022) whose momentum was 0.999 and learning rate of 1e-5 was adopted. Besides, the kernel size was set to (3,3), and the dropout rate was set to 0.1. The validation loss was monitored at every epoch and the best weight of the model would be saved when the validation loss is smallest in the iterative process.

### 3.1 Evaluation metrics

To evaluate the performance of the PDC-Net on the dataset, Sensitivity (Shen et al., 2022; Siar and Teshnehlab, 2022), Specificity (Rai and Chatterjee, 2021; Ramachandran et al., 2021), Dice value (Yang Y et al., 2020; You et al., 2021), and Intersection over Union (Ahmed et al., 2021; Zou et al., 2021) are utilized as evaluation metrics, which can be defined as:

$$Sensitivity=\frac{TP}{TP+FP}$$ (2)

$$Specificity=\frac{TN}{FP+TN}$$ (3)

$$Dice=\frac{2TP}{2TP+FN+FP}$$ (4)

$$IoU=\frac{TP}{TP+FN+FP}$$ (5)

where TP represents the number of adenoma pixels judged to be adenoma, TN represents the number of non-adenoma pixels judged to be non-adenoma, FP represents the number of non-adenoma pixels judged to be adenoma, and FN represents the number of adenoma pixels judged to be non-adenoma.

### 3.2 Results of PDC-Net

**FIGURE 6:**
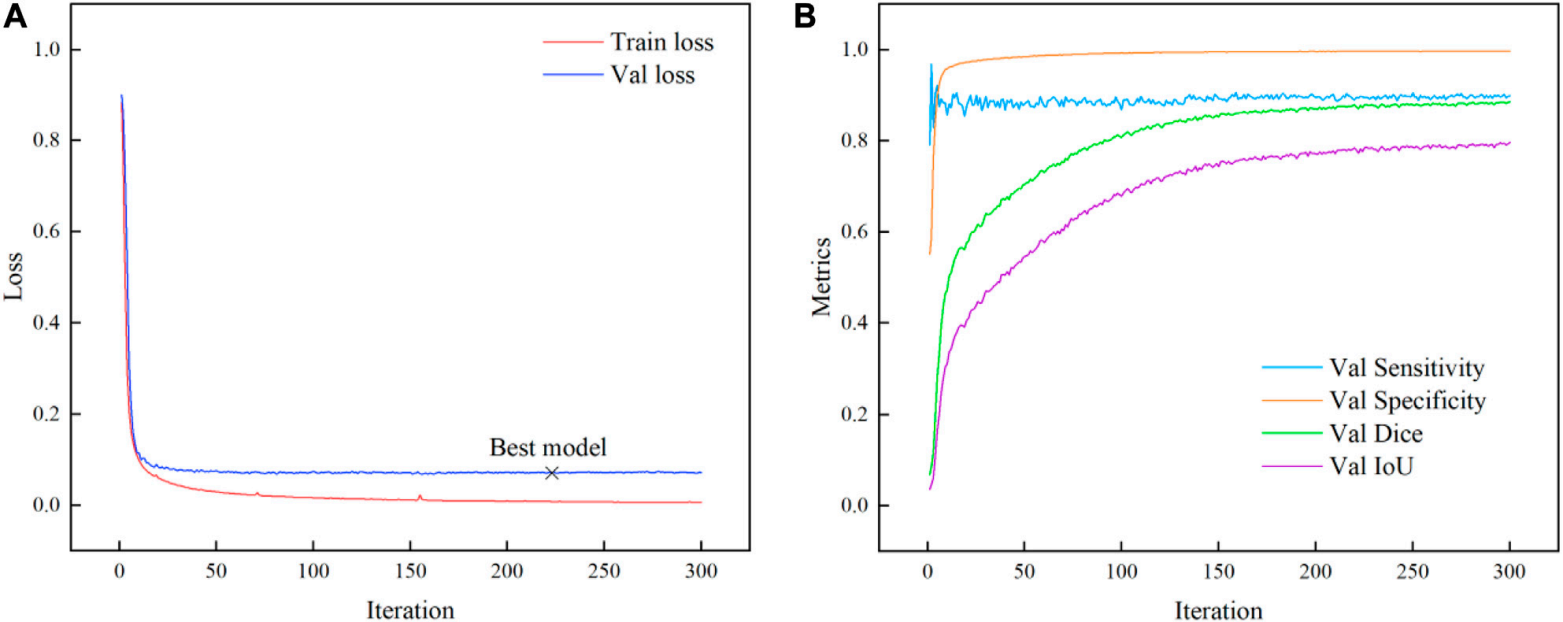
Iterative process parameters change. **(A)** Loss. **(B)** Evaluate metrics.

As shown in Figure 6A, the change in loss value during training is demonstrated. After 220 steps, although the loss on the training set is in a slow decline state, the loss on the validation set gradually tends to be flat or even rises slowly, which indicates that the optimal weight can be obtained when the number of iterations of the model is set at 300 steps. Finally, the model obtained 0.9092, 0.9968, 0.8845, 0.7943 on the four metrics of Sensitivity, Specificity, Dice and IoU, respectively. The iterative change of evaluation metrics is shown in Figure 6B.

**FIGURE 7:**
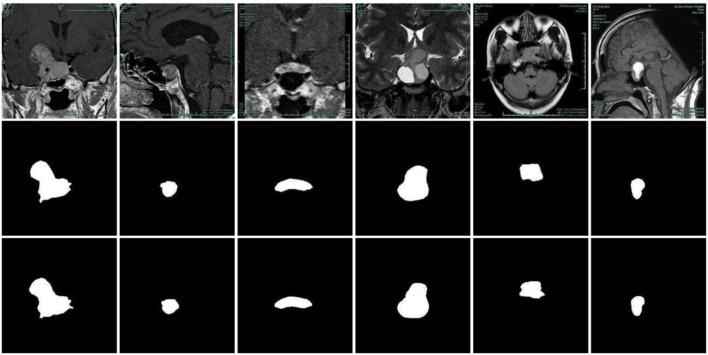
Pituitary adenoma image segmentation results of PDC-Net model. The first row: original images. The second row: the gold standard sketched by experts. The third row: results of PDC-Net.

The example of the results of the PDC-Net model on the pituitary adenoma segmentation dataset is illustrated in Figure 7. The results show that the proposed model can segment the boundaries of pituitary adenomas from brain MR Images through different angles. Although the labelling process is carried out by several experts and reviewed repeatedly, it is difficult to accurately mark manually with human eyes. It is gratifying that for some segmentation results, PDC-Net is more accurate than manual labeling results, as shown in the third row of Figure 7.

### 3.3 Ablation experiment

To verify the function of the suggested module and the associated structure, ablation experiments were conducted, and the relevant conclusions were discussed as follows.

#### 3.3.1 PDCM module

To expand the receptive field, raise the capacity of the model to extract multi-scale information, and improve the network segmentation accuracy, the PDCM was proposed. The proposed PDCM modules are of two types, one without the previous branch and the other is the fusion of previous branch features (PDCM). As shown in Table 1, the segmentation results of several structures are presented. The results show that the model with the previous branch features has a slight improvement in every metric compared with the model without previous branch features. It is worth noting that the model incorporating small receptive field features has relatively large improvements in each evaluation metric.

**TABLE 1:** Ablation experiment of the PDCM module.

	Sensitivity	Specificity	Dice	IoU
No PDCM	0.8949	0.9965	0.8696	0.7721
PDCM without previous branch	0.8993	0.9966	0.8705	0.7722
PDCM with previous branch	0.9092	0.9968	0.8845	0.7943

**FIGURE 8:**
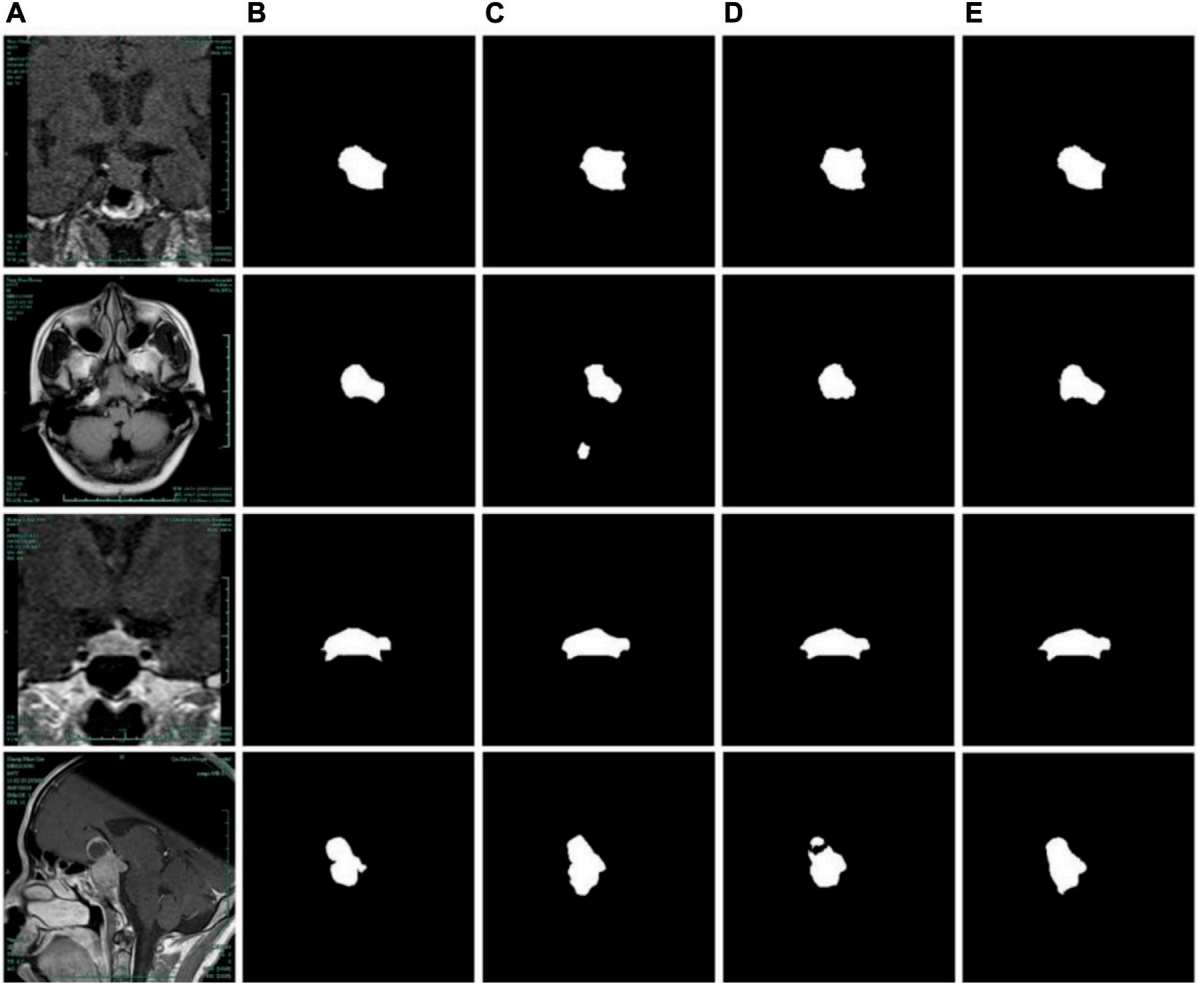
Comparison of segmentation results of different PDCM modules. **(A)** Original images. **(B)** Label images. **(C)** No PDCM. **(D)** PDCM without previous branch. **(E)** PDCM with previous branch.

As displayed in Figure 8, the segmentation results of the three structures in Table 1 are presented. By comparing the results in the second row of Figure 8, it can be found that the model without the PDCM module does not understand the global information enough, leading to false detection of the non-adenoma region. Although the simple PDCM connection concatenates the convolution results of different receptive fields, it lacks the connection between small receptive fields and large receptive fields. PDCM with previous branch can solve this problem well, as shown in Figures 8D, E.

#### 3.3.2 Residual connection

For deep learning, simply increasing the depth of the network to improve the learning ability of the network often leads to the problem of gradient disappearance. The feature of the residual network is to improve the learning ability and accuracy of the deep network by adding jump connections. As shown in Table 2, ablation experiments concerning residual connection were presented. The results show that when the PDCM module was not added, the addition of residual gave a small boost to the model due to the relatively shallow network. When PDCM with previous branch was added, the model with residual was improved by more than one percentage point in Dice and IoU due to the relatively deep network.

**TABLE 2:** Ablation experiment of residual connection.

	Sensitivity	Specificity	Dice	IoU
No Residual + No PDCM	0.8827	0.9963	0.8655	0.7660
Residual + No PDCM	0.8949	0.9965	0.8696	0.7721
No Residual + PDCM with previous branch	0.9077	0.9964	0.8762	0.7810
Residual + PDCM with previous branch	0.9092	0.9968	0.8845	0.7943

**FIGURE 9:**
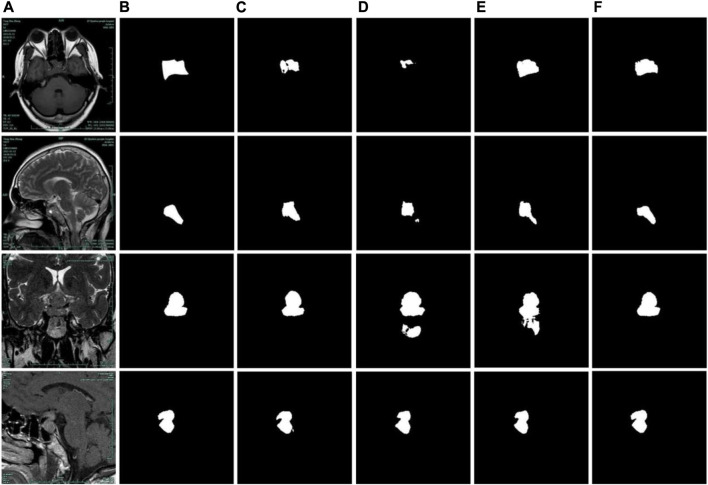
Comparison of segmentation results of residual structures. **(A)** Original images. **(B)** Label images. **(C)** No Residual + No PDCM. **(D)** Residual + No PDCM. **(E)** No Residual + PDCM with previous branch. **(F)** Residual + PDCM with previous branch.

The segmentation results concerning the Residual structure were shown in Figure 9. As shown in Figures 9C, D, for the network with PDCM structure, the addition of residual structure affects the segmentation accuracy of the adenoma boundary, even if there is a slight improvement in the evaluation metrics. On the contrary, when the PDCM with previous branch structure is added to the network, the learning ability of the model is reduced due to the too-deep structure, as shown in Figure 9E. At this time, the addition of residual can effectively improve the segmentation accuracy of the model and provide a more accurate description of the adenoma boundary.

### 3.4 Comparison with different models

To evaluate the performance of the model more comprehensively, the proposed model is compared with some classical and newly proposed models, and the evaluation metrics are shown in Table 3. The results illustrate that the performance of the proposed PDC-Net on the pituitary adenoma dataset is better than other models involved in the comparison.

**TABLE 3:** The results of comparison with other models.

	Sensitivity	Specificity	Dice	IoU
U-Net (Ronneberger et al., 2015)	0.8821	0.9868	0.8661	0.7715
AttUNet (Oktay et al., 2018)	0.8904	0.9965	0.8683	0.7802
SegNet (Badrinarayanan et al., 2017)	0.8689	0.9961	0.8680	0.7695
ODSegmentation (Wang J K et al., 2021)	0.8899	0.9968	0.8735	0.7771
CLNet (Zheng et al., 2021)	0.8860	0.9966	0.8679	0.7685
MMDC-UNet (Jin et al., 2022)	0.8777	0.9966	0.8828	0.7927
DeepLabV3+ (Chen et al., 2018a)	0.8930	0.9961	0.8618	0.7589
SK-UNet (Byra et al., 2020)	0.9091	0.9963	0.8736	0.7775
SAR-UNet (Wang L et al., 2021)	0.8850	0.9926	0.7936	0.6613
PDC-Net	0.9092	0.9968	0.8845	0.7943

**FIGURE 10:**
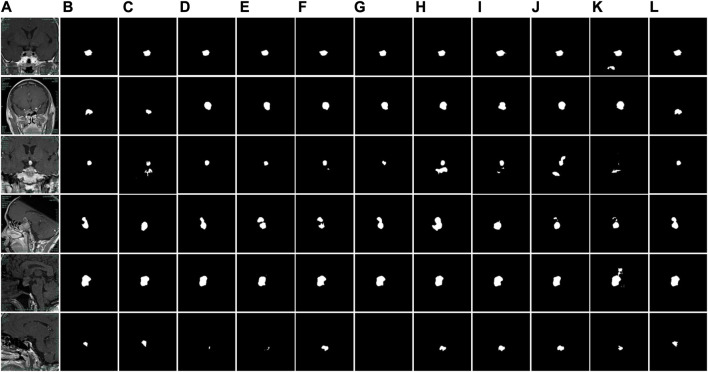
Comparison of segmentation results of different models. **(A)** Original images. **(B)** Label images. **(C)** U-Net. **(D)** AttUNet. **(E)** SegNet. **(F)** ODSegmentation. **(G)** CLNet. **(H)** MMDC-Net. **(I)** DeepLabV3+. **(J)** SK-UNet. **(K)** SAR-UNet. **(L)** PDC-Net.

The segmentation results of different models are illustrated in Figure 10. Despite the fact that the site of the pituitary adenoma varies for distinct brain MR images, the location is almost certain for brain MR images taken in the same direction. Due to the lack of correlation between high and low semantic features, U-Net will produce missing pixels when facing some dermoscopic images with large background interference and complex color changes, as shown in Figure 10C. Since the attention mechanism is more for the localization of adenomas and lacks the connection of high and low-scale features, the network with the attention mechanism cannot better complete the description of the boundary contour, as shown in Figures 10D, J, K. Some network architectures cannot produce better results on the pituitary adenoma data set because of the complicated network connections and inappropriate depth of the network, as illustrated in Figures 10F–I. The experimental results show that the proposed network structure can accurately segment pituitary adenomas from brain MR images in all directions, which has important research significance for intelligent and automatic analysis of medical images.

## 4 Conclusion

Taking pituitary adenomas as the research object, this study constructed a deep learning model based on U-Net architecture to segment the boundaries of adenomas, and the relevant conclusions were as follows: firstly, the proposed PDCM module with previous branch can effectively combine the feature information of different scales and describe the adenoma boundary more accurately. Secondly, the integration of residual connection makes the deep network structure combined with the PDCM module obtain stronger learning ability and improves the performance of the model on the dataset. The results show that the proposed method has research significance for the automatic and intelligent detection of pituitary adenoma. Finally, the proposed PDC-Net can accurately segment pituitary adenomas from brain MR images, and it determines 90.92% of Sensitivity, 99.68% of Specificity, 88.45% of Dice value and 79.43% of Intersection over Union.

## Data Availability

The original contributions presented in the study are included in the article/supplementary material, further inquiries can be directed to the corresponding authors.
